# An experimentally-supported genome-scale metabolic network reconstruction for *Yersinia pestis *CO92

**DOI:** 10.1186/1752-0509-5-163

**Published:** 2011-10-13

**Authors:** Pep Charusanti, Sadhana Chauhan, Kathleen McAteer, Joshua A Lerman, Daniel R Hyduke, Vladimir L Motin, Charles Ansong, Joshua N Adkins, Bernhard O Palsson

**Affiliations:** 1 Department of Bioengineering, University of California, San Diego, La Jolla, California, USA; 2 Departments of Microbiology and Immunology, University of Texas Medical Branch, Galveston, Texas, USA; 3 Biological Sciences Division, Pacific Northwest National Laboratory, Richland, Washington, USA; 4 Department of Pathology, University of Texas Medical Branch, Galveston, Texas, USA

## Abstract

**Background:**

*Yersinia pestis *is a gram-negative bacterium that causes plague, a disease linked historically to the Black Death in Europe during the Middle Ages and to several outbreaks during the modern era. Metabolism in *Y. pestis *displays remarkable flexibility and robustness, allowing the bacterium to proliferate in both warm-blooded mammalian hosts and cold-blooded insect vectors such as fleas.

**Results:**

Here we report a genome-scale reconstruction and mathematical model of metabolism for *Y. pestis *CO92 and supporting experimental growth and metabolite measurements. The model contains 815 genes, 678 proteins, 963 unique metabolites and 1678 reactions, accurately simulates growth on a range of carbon sources both qualitatively and quantitatively, and identifies gaps in several key biosynthetic pathways and suggests how those gaps might be filled. Furthermore, our model presents hypotheses to explain certain known nutritional requirements characteristic of this strain.

**Conclusions:**

*Y. pestis *continues to be a dangerous threat to human health during modern times. The *Y. pestis *genome-scale metabolic reconstruction presented here, which has been benchmarked against experimental data and correctly reproduces known phenotypes, provides an *in silico *platform with which to investigate the metabolism of this important human pathogen.

## Background

*Yersinia pestis *is a gram-negative bacterium within the family Enterobacteriaceae that, along with *Yersinia pseudotuberculosis *and *Yersinia enterocolitica*, is one of three members of its genus that can cause disease in humans. *Y. pestis *diverged from *Y. pseudotuberculosis *only 1,500 - 20,000 years ago, but *Y. pestis *and *Y. pseudotuberculosis *diverged from *Y*. *enterocolitica *in the more distant past [1]. Despite their close evolutionary relationship, the diseases they cause differ markedly. Whereas *Y. pseudotuberculosis *and *Y. enterocolitica *are primarily gastrointestinal pathogens in humans, *Y. pestis *infections lead to a systemic disease known as plague that can become fatal rapidly. In the last 2000 years, there have been three distinct outbreaks of plague that have led to a large number of fatalities: the Justinian plague between the 5^th ^and 7^th ^centuries, the Black Death in Europe between the 13^th ^and 15^th ^centuries, and the modern plague from the latter half of the 1800s to the present. These outbreaks of fatal infections that continue to occur [2,3], the recent isolation of a multi-drug resistant strain [4], and the potential to develop into a bioweapon all signify that *Y. pestis *remains a significant threat to human health.

Most *Y. pestis *strains can be divided into three biovars: Antigua, Mediavalis, and Orientalis, based on their ability to ferment glycerol and reduce nitrate [5]. Biovar Antigua strains can do both; biovar Mediavalis strains can ferment glycerol but cannot reduce nitrate; and biovar Orientalis strains cannot ferment glycerol but can reduce nitrate [5]. Moreover, each biovar has been linked to one of the three pandemics. Biovar Antigua is associated with the Justinian plague; biovar Mediavalis is associated with the Black Death; and biovar Orientalis is associated with modern plague [5]. Debate surrounding these associations continues, however, as recent data suggest that the Black Death might not have been caused by strains belonging to Biovar Mediavalis but rather by at least two strains that pre-date the emergence of and are distinct from both biovar Mediavalis and biovar Orientalis [6].

Here we present a metabolic network reconstruction and corresponding mathematical model of metabolism in *Y. pestis *CO92 (abbreviated YP CO92). This strain is classified within Biovar Orientalis and is virulent to humans, a feature which distinguishes the model presented here from one published previously for *Y. pestis *91001 [7], which is avirulent to humans. We also present a comprehensive analysis of pathways in the metabolic network related to the biosynthesis and utilization of key metabolites, acquire experimental data from growth on several different carbon sources and compare them to simulation results, and identify essential genes that might constitute possible targets for antibiotic development.

## Results

### Characteristics of the reconstruction

**Figure 1:**
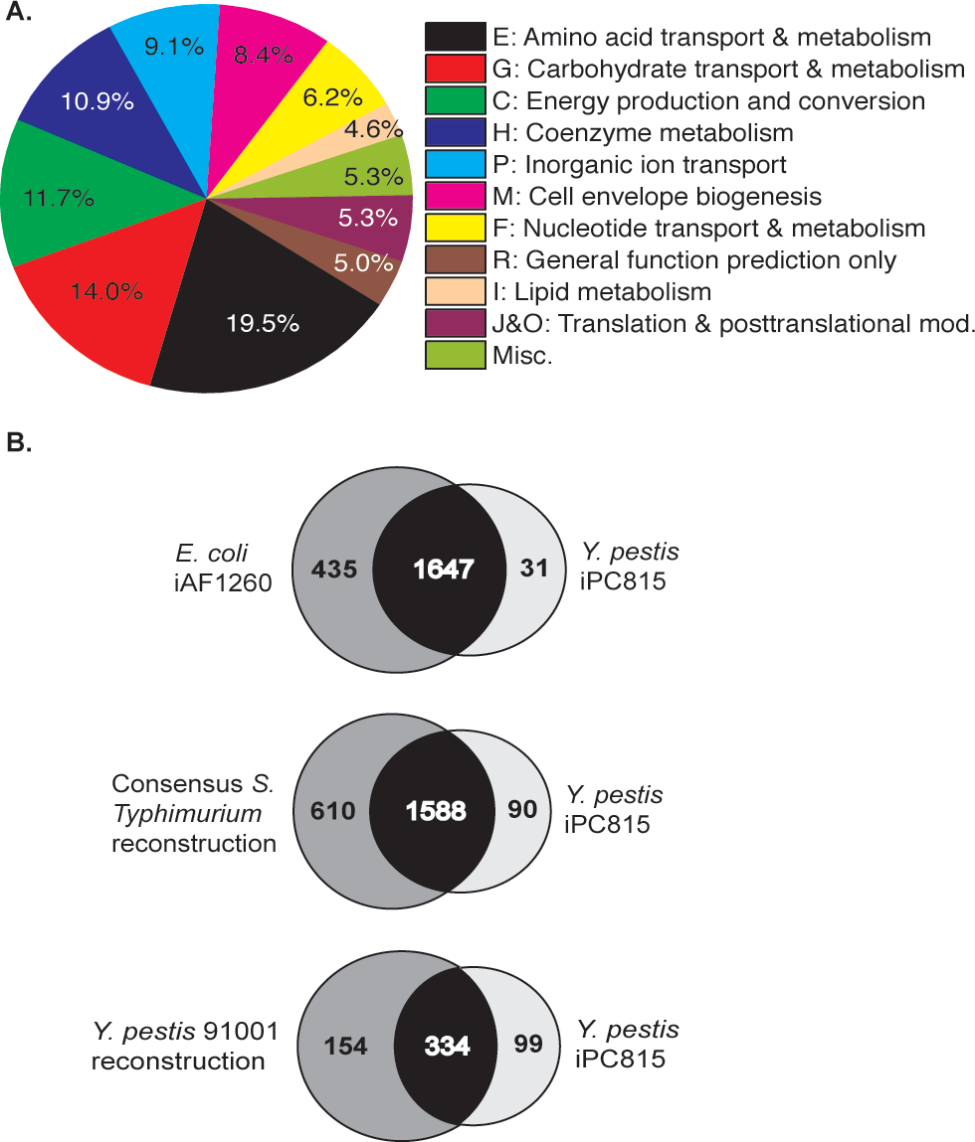
**A. Breakdown of the genes in iPC815 by COG classification**. The COG classifications that make up the Miscellaneous category are: K, L, Q, S, T, U, and V. **B**. Venn diagrams showing the overlap between iPC815 and published *E. coli*, *S*. Typhimurium, and *Y. pestis *91001 reconstructions based on shared versus organism-specific reactions (*E. coli *and *S*. Typhimurium) and E.C. numbers (*Y. pestis *91001). In the latter case, an E.C. number associated with more than one reaction was only counted once. The low overlap between iPC815 and the *Y. pestis *91001 reconstruction arises from the use of E.C. numbers to compare the two models rather than true biological differences between the two strains.

The *Y. pestis *CO92 metabolic reconstruction presented here, iPC815, contains 815 genes, 678 proteins, 963 unique metabolites, and 1678 reactions. iPC815 was assembled by comparison to existing *E. coli *[8], *Salmonella enterica *serovar Typhimurium (hereafter referred to as *S*. Typhimurium) [9], and *Y. pestis *strain 91001 [7] metabolic reconstructions; genetic, protein, metabolite, and biochemical data contained in databases such as KEGG [10], BRENDA [11], MetaCyc [12], and PATRIC [13]; and genus-, species-, and strain-specific information gleaned from the literature. The model contains distinct compartments for the cytoplasm and periplasm, and inner and outer membrane transporters and their associated transport reactions model the movement of metabolites between the compartments. Of the 1678 reactions, 1539 (92%) are linked to their corresponding genes and proteins through gene-protein-reaction associations (GPRs); the majority of the 139 non-linked reactions are transport reactions. Table 1 summarizes the general features of iPC815, while Figure 1 depicts the functional classification of the 815 genes according to their COG function and presents a comparison to the *E. coli, S*. Typhimurium LT2, and *Y. pestis *91001 metabolic reconstructions. The full list of all genes, metabolites, reactions, and GPRs in iPC815 can be found in Additional File 1.

**Table 1:** Overview of iPC815.

Category	Subsystem	Number
**Genes**		815
**Proteins**		678
**Metabolites**		
	Cytoplasm	873
	Periplasm	398
	External	281
	Unique	963
	Total	1552
**Metabolic Reactions**		
	Cytoplasm	932
	Periplasm	133
	GPR-associated	1539
	Non-GPR-associated	139
	Unique	1678
**Transport Reactions**		
	Periplasm <=> External	281
	Cytoplasm <=> Periplasm	332
**Exchange Reactions**		281
**Biomass Objective Functions**		2
**Total Reactions**		1961

To compile the number of unique metabolites, identical compounds appearing in more than one compartment were only counted once. Unique metabolic reactions encompass all reactions except exchange reactions and biomass objective functions.

The iPC815 model contains two biomass objective functions (BOFs) [14] that were derived from the two BOFs developed for the existing *Y. pestis *91001 reconstruction [7] and modified as follows. First, iPC815 contains pathways for the biosynthesis of four *Y. pestis *fatty acid acyl chains that are not explicitly modeled in the *Y. pestis *91001 reconstruction, specifically 14:0, 16:1, 16:0, and 18:1. These four comprise approximately 86% of the total fatty acid composition in 29 *Y. pestis *isolates during growth at 28°C [15]. Second, the lipopolysaccharide (LPS) core oligosaccharide contains an uncommon D-glycero-D-talo-oct-2-ulosonic acid (Ko) analog not normally found in Gram-negative bacteria [16,17]. Both of these features have been incorporated into the two BOFs in iPC815.

Two BOFs are needed because the *Y. pestis *biomass composition differs at 25-28°C versus 37°C. The significance of these two temperatures stems from the two types of hosts that *Y*. *pestis *infects in the natural environment: insect vectors at ambient temperature and mammalian hosts with regulated body temperatures of about 37°C. Accordingly, most laboratory studies of *Y. pestis *are carried out at one or both of these temperatures, and data from such studies have revealed two principal differences in biomass composition at 25-28°C versus 37°C. The first involves the lipopolysaccharide (LPS) structure: at 37°C the LPS is composed of one predominant core oligosaccharide and one predominant lipid A isoform (tetraacyl 14:0), whereas at 25°C the LPS is composed of multiple structures for both [17]. The second involves the fatty acid composition. Lipid measurements from *Y. enterocolitica *indicate that the abundance of various fatty acids varies with growth temperature [18], and we assume that this characteristic holds for *Y. pestis *as well. The coefficients of the terms representing the four fatty acids consequently differ between the two BOFs. The 25-28°C BOF covers a temperature range rather than a single value since the experimental data on which this BOF was constructed were taken over this range. Thus, the model also assumes that the BOF composition does not change appreciably between 25°C and 28°C.

### Gap analysis of biosynthetic pathways

There were a number of gaps in iPC815 that initially prevented the simulation of biomass formation. These gaps consisted of reactions that were essential for simulating growth but for which we could not identify homologs in YP CO92 to the gene products in *E. coli*, *S*. Typhimurium, or *Y. pestis *91001 that catalyze the same reactions. Each of these gaps constitutes testable experimental hypotheses.

**Figure 2:**
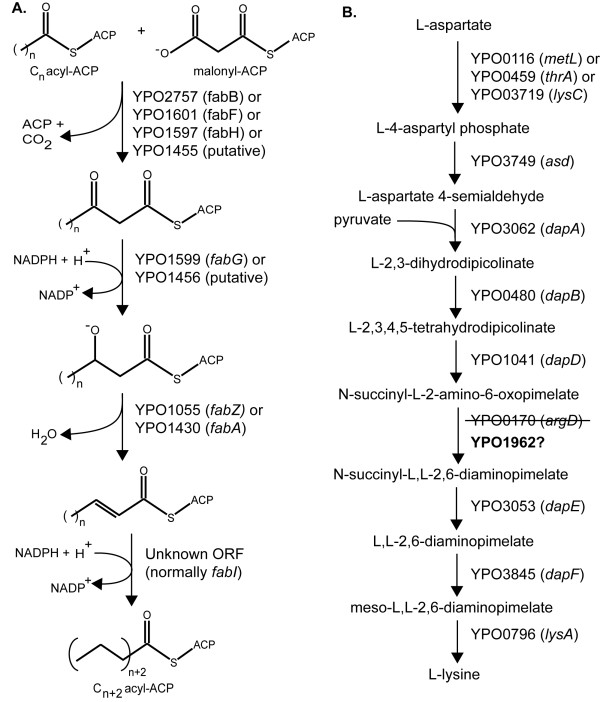
**Essential gaps in key *Y. pestis *CO92 metabolic pathways**. **A**. The last step during each fatty acid elongation cycle involves the reduction of an enoyl-ACP intermediate. In other Enterobacteria such as *E. coli *and *S*. Typhimurium, FabI catalyzes this step; however, the current annotation of the YP CO92 genome does not contain *fabI*. **B**. The enzyme ArgD catalyzes the sixth step in the nine-step lysine biosynthesis pathway, but *argD *(YPO0170) is disrupted in YP CO92. YPO1962 has the greatest homology to YPO0170 within the *Y. pestis *CO92 genome, and we hypothesize here that YPO1962 can replace the function of YPO0170. Note: although each reaction in each pathway is depicted as unidirectional, some are reversible.

One gap occurs in the fatty acid biosynthesis pathway (Figure 2A). The current annotation of the YP CO92 genome indicates that this strain contains the same full suite of genes for fatty acid biosynthesis that *E. coli *and *S*. Typhimurium have except one, the enoyl acyl-carrier protein (ACP) reductase *fabI*. The enzyme encoded by *fabI *catalyzes the reduction of the 2,3 double bond in diverse acyl units during chain elongation [19], which is an essential step in fatty acid biosynthesis. Consistent with this data, the model cannot simulate growth without this key enzyme. We have therefore included this reaction in the model but it is not associated with a gene or protein.

A similar gap appears in the lysine biosynthesis pathway. Lysine is synthesized from aspartate in both *E. coli *and *S*. Typhimurium through a series of nine enzyme-catalyzed reactions [20], and YP CO92 has homologs to each of the nine enzymes except one, ArgD (YPO0170) (Figure 2B). In YP CO92, an IS element (*IS100*) is inserted directly into the ORF of YPO0170 [21], splitting it into two and likely rendering it nonfunctional. Despite this disruption, we experimentally tested and found no evidence for lysine auxotrophy in YP CO92, a phenotypic observation that suggests additional enzyme(s) can catalyze the same reaction as YPO0170 during lysine biosynthesis. We consequently searched for alternative enzymes within the YP CO92 genome that can accept both N-succinyl-L-2-amino-6-oxopimelate and any molecule capable of donating an amino group as reactants (Figure 2B), but found no support for such an enzyme. Subsequently, we searched for paralogs within the YP CO92 genome that have homology to both YPO0170 fragments. The gene with the greatest homology was YPO1962 with 59% nucleotide identity, and we have assumed in the model that YPO1962 can replace YPO0170 in the lysine biosynthetic pathway (Figure 2B).

### Phenotype simulations

**Figure 3:**
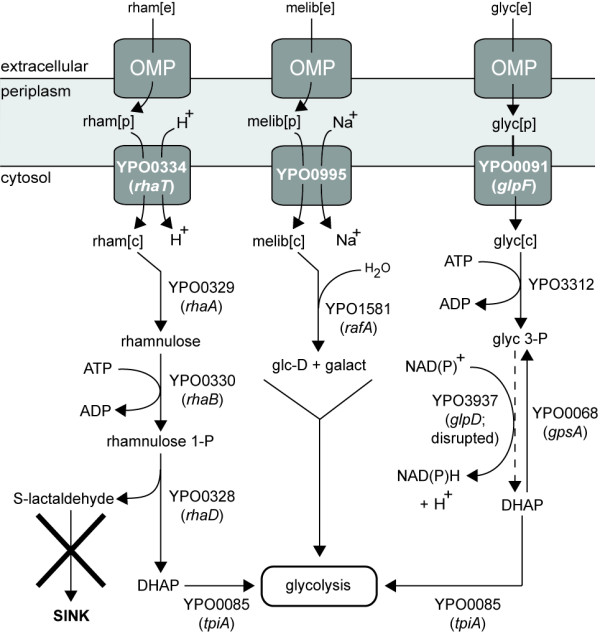
**Analysis of possible defects within the *Y. pestis *CO92 rhamnose, melibiose, and glycerol uptake pathways**. Epidemic *Y. pestis *strains such as CO92 cannot utilize rhamnose, and YP CO92 cannot ferment glycerol. The dashed line indicates a reaction catalyzed by GlpD (YPO3937) that is present in most other Enterobacteria but appears to be absent in YP CO92 due to a disruption in an underlying gene. Abbreviations: rham, rhamnose; 1-P, 1-phosphate; 3-P: 3-phosphate; DHAP, dihydroxyacetone phosphate; melib, melibiose; glc-D, D-glucose; galact, galactose; glyc, glycerol; OMP, outer membrane porin. The suffixes [e], [p], and [c] denote metabolites located in extracellular, periplasmic, and cytoplasmic compartments, respectively.

Once all critical gaps necessary for *in silico *biomass formation were filled, we deployed the model to compute growth versus no-growth on different carbon, nitrogen, phosphorus, and sulfur sources at both 25-28°C and 37°C (Additional File 1). We focused particular attention on rhamnose and melibiose since the ability or inability to ferment these two sugars is one of several phenotypes commonly used to classify different strains of *Y. pestis*. For example, two of the best predictors for virulence in humans are the absence of rhamnose fermentation and virulence in guinea pigs [22]. YP CO92 is known to be lethal to humans, and consistent with this phenotype there is no growth *in silico *if rhamnose is the sole carbon source. The model predicts that this outcome is due to the overaccumulation of L-lactaldehyde. Specifically, the uptake pathway that connects periplasmic rhamnose with central metabolism (Figure 3) leads to the synthesis of a byproduct, L-lactaldehyde, that is not removed through secretion, degradation, incorporation into metabolism, or by any other means. Its production via this pathway would therefore result in an infinite accumulation of the compound, which is a thermodynamically impossible outcome. On the other hand, the melibiose utilization pathway appears to be intact and thermodynamically consistent. We assume that a general outer membrane porin allows passage of melibiose from the extracellular space into the periplasm, after which an inner membrane sodium:galactoside symporter (YPO0995) appears capable of transporting melibiose into the cytosol. Subsequently, the alpha-galactosidase RafA (YPO1581) [23] connects imported melibiose with central metabolism (Figure 3). This analysis implies that regulation of these genes in the melibiose utilization pathway, rather than the genes themselves, underlies the inability of YP CO92 to utilize melibiose. We accounted for this observation by retaining this set of reactions in the model but deactivating the melibiose extracellular to periplasm uptake reaction.

Using the individual components of the BCS chemical medium to constrain the model [24], we next assessed whether the model could recapitulate common amino acid auxotrophies seen in epidemic strains of *Y. pestis *such as CO92. These are methionine, phenylalanine, and the combination of glycine and threonine [25,26]. Consistent with these data, the model exhibits auxotrophy for methionine and phenylalanine but growth still occurs at a slow rate (~0.08 hr^-1^) *in silico *in the absence of both glycine and threonine as long as all other amino acid components in the BCS medium are present. Prior experimental studies have reported the isolation of *Y*. *pestis *clones that grow in media lacking the two amino acids after several days incubation and attributed the mechanism to meiotrophy [25]. We observed identical behavior when we experimentally tested for glycine/threonine auxotrophy in YP CO92: we could detect growth one day after inoculating it into media supplemented with threonine, but glycine/threonine-deficient media did not support growth until several days after inoculation. Simulation results, however, suggest that slow growth rather than meiotrophy might be sufficient to explain the glycine/threonine phenotype. The model does not exhibit any other amino acid auxotrophy.

To further evaluate the performance of the model against experimental data, we tested whether YP CO92 could grow on eleven different carbon sources and compared the data against simulation results. Initially, model simulations correctly reproduced the experimental data for ten of the eleven compounds (Table 2). Glycerol was the only exception: the simulations originally permitted growth on glycerol but no growth was seen when YP CO92 was cultured in media containing glycerol as the principal carbon source, a result that is consistent with prior studies of YP CO92 [27]. It has been proposed that a 93 bp deletion in *glpD *(YPO3937), which encodes aerobic glycerol 3-phosphate dehydrogenase, might render the protein nonfunctional and account for this growth phenotype [27]. There is a second glycerol 3-phosphate dehydrogenase in the YP CO92 genome, *gpsA *(YPO0068); however, the GpsA homolog in *E*. *coli *only catalyzes the unidirectional conversion of dihydroxyacetone phosphate to glycerol 3-phosphate (Figure 3). The protein cannot catalyze the reverse reaction, which is the necessary direction for glycerol assimilation [28]. These data were subsequently incorporated into the model to make it more consistent with the observed glycerol-negative phenotype, but slow growth (0.07 hr^-1^) still occurs *in silico *despite these modifications because the model permits glycerol to enter central metabolism indirectly through the fatty acid biosynthesis and degradation pathways. Although not tested here, lactose represents another discrepancy between model simulations and experimental data (Additional File 1). None of the three pathogenic Yersiniae can metabolize lactose, but model simulations erroneously suggest that YP CO92 can.

**Table 2:** Comparison between experimental data and model simulations for growth on eleven different carbon sources.

Carbon Source	Experimental	Computational	Comments
Gluconate	Yes (0.27)	Yes	
		Without citrate: 0.27	
		With citrate: 0.28	
Glucose	Yes (0.29)	Yes	
		Without citrate: 0.28	
		With citrate: 0.28	
Glycerol	No	Yes	Model suggests glycerol can enter central metabolism indirectly through fatty acid biosynthesis and degradation pathways, thereby permitting slow growth
Acetate	Yes (0.14)	Yes	
Citrate	Yes (0.086)	Yes	
Lactate	Yes (0.12)	Yes	
Xylose	Yes (0.20)	Yes	
Ribose	Yes (0.17)	Yes	
Galactose	Yes (0.16)	Yes	
Maltose	Yes (0.27)	Yes	
Arabinose	Yes (0.28)	Yes	

Values in parentheses in the Experimental column are the measured growth rates performed as part of this study. Values in parentheses in the Computational column indicate model-predicted growth rates in BCS defined media with or without citrate and with glucose or gluconate as the principal carbon source.

For two of the eleven compounds (glucose and gluconate), we also measured the uptake and secretion rates for a set of metabolites during growth at 26°C, constrained the model according to the data (Additional File 1), and assessed the ability of the constrained model to accurately compute growth rates on the two carbon sources. Both growth media contained citrate in addition to the two carbon sources; citrate is a common component of the BCS medium routinely used to culture *Yersinia *spp [24]. The simulated and experimental growth rates agreed well on both sugars (Table 2) regardless of the presence or absence of citrate in the simulation, which suggests that YP CO92 utilizes negligible amounts of citrate when either glucose or gluconate are present.

### Gene Essentiality

**Figure 4:**
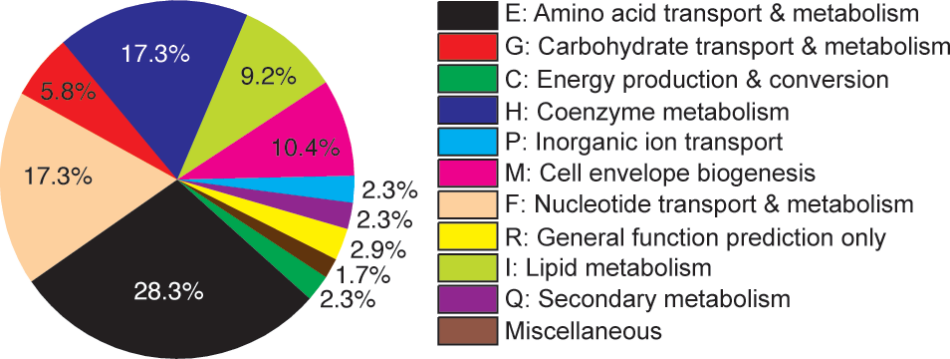
**Breakdown by COG classification of 146 genes predicted to be essential in *Y. pestis *CO92**. The 146 genes cover growth at both 25-28°C and 37°C and on both glucose and gluconate defined media (Additional File 2). The COG classifications that make up the Miscellaneous category are: S, V, and Unassigned.

We used the reconstruction to predict essential metabolic genes in YP CO92 for growth on the same glucose and gluconate defined media we used to culture the bacterium (Figure 4). In total, 142 identical genes are predicted to be essential on both media and at both growth temperatures. An additional three genes, YPO1139, YPO2063, and YPO3632, are predicted to be essential at 25-28°C only. These three genes are involved in the biosynthesis of two forms of lipid A that are present only at 25-28°C [17]. A fourth gene, YPO3718 (*pgi*), is predicted to be essential on gluconate only because its deletion disrupts gluconeogenesis and consequently the biosynthesis of glycogen, an indispensable component of the YP CO92 biomass objective function. The full list of genes predicted to be essential on the two media can be found in Additional File 2.

The largest group of predicted essential genes falls within amino acid transport and metabolism (28.3%). Removal of genes in this subset imitates one of the three amino acid auxotrophies that characterize this strain. The next largest groups are nucleotide and coenzyme metabolism (17.3% each) followed by cell membrane biogenesis (10.4%) and lipid metabolism (9.2%); however, many genes in these groups were also deemed to be essential according to the *E. coli *and *S*. Typhimurium metabolic reconstructions on which many of the gene-protein-reactions in iPC815 are based [8,9] (Additional File 2). This set of predicted essential genes therefore reflects probable overlap among the three models. In turn, the overlap arises from what is likely common biology that is shared within the family of *Enterobacteriaceae*. It might be possible to exploit this feature for the development of broad-spectrum antibiotics targeted against this group of human pathogens.

## Discussion

Metabolic reconstructions constitute a framework to organize genomic, proteomic, metabolomic, and other data sets and to assess the effects of perturbations on these elements at the network level. Accordingly, we present a metabolic reconstruction for YP CO92, a strain that is virulent to humans, and benchmark the reconstruction qualitatively against experimental growth data from eleven different carbon sources and quantitatively against growth rate and metabolite uptake and secretion rate data from two of the sources.

The two gaps in lysine and fatty acid biosynthesis highlighted in this work are significant because model simulations cannot occur unless both gaps are filled. The gap in lysine biosynthesis is noteworthy in that the disrupted gene, YPO0170, encodes a bifunctional enzyme, ArgD, that is involved in arginine biosynthesis as well [29]: specifically, ArgD catalyzes the reversible conversion of N-acetyl-L-glutamate 5-semialdehyde and L-glutamate to N-acetyl-L-ornithine and 2-oxoglutarate, after which N-acetyl-L-ornithine is converted to L-ornithine. An absence of ArgD does not result in arginine auxotrophy, however, because YP CO92 contains an alternative, one-step route to L-ornithine from L-proline that is catalyzed by YPO4090. This reaction is included in the model. On the other hand, there is no clear paralog or other mechanism within YP CO92 that appears capable of replacing the missing *fabI *gene in fatty acid biosynthesis. At the amino acid level, there is only 28% identity between *E. coli *FabI and its best match in YP CO92, YPO3351. We therefore sought to identify possible candidate enzymes through an analysis of expression data for YP CO92 [Schrimpe-Rutledge AC and Adkins JN, unpublished data], reasoning that the unidentified gene will be located near other genes involved in fatty acid biosynthesis (specifically, the cluster from YPO1597 (*fabH*) through YPO1601 (*fabF*)), show correlated expression with the genes in this cluster, and be annotated as hypothetical. The best match based on these criteria is the hypothetical gene YPO1594. Consequently, we propose that YPO1594 might possess the ability to carry out the same catalytic function as FabI. Other genes showing correlated expression but located farther away from YPO1597-YPO1601 are YPO3732 and YPO2055.

Pathway analysis and model simulations led to the hypothesis that YP CO92 cannot utilize rhamnose and melibiose due to a missing sink reaction and perturbed gene regulation, respectively, but other possible mechanisms have been advanced. A disruption in gene regulation might contribute to the rhamnose-negative phenotype as well. Specifically, a recent study that compared the sequences of rhamnose fermentation genes in rhamnose-positive and rhamnose-negative strains suggested that a point mutation in the transcriptional activator RhaS might be responsible for the rhamnose-negative phenotype [30]. Experimental data from a prior study indirectly support this claim [31]. Rhamnose-negative strains can revert and gain the ability to metabolize the sugar at a low frequency, and an analysis of one such strain found that RhaB and RhaA had become active in the revertant [31]. Since RhaS regulates the rhaBAD operon [32], these data imply that RhaS is the key regulatory protein controlling rhamnose utilization and that the mechanism involves rhaBAD. On the other hand, melibiose metabolism might be absent in YP CO92 because of one or more defects in inner membrane transport. We identified two possible melibiose symporters in YP CO92, YPO1582 and YPO0995 (*melB*). The former is the homolog of the putative melibiose symporter YP1470 from *Y. pestis *91001, a strain that can utilize melibiose [33], but the 5' end of the coding sequence for YPO1582 has been disrupted by the IS element IS285. YPO1582 is therefore presumed to be a pseudogene. The latter is intact in YP CO92; however, the IS element IS1661 is located approximately 250 bp upstream from the 5' end of *melB*, raising the possibility that it has potentially disrupted the control of expression of *melB*. Proteomic data collected during mid-log growth on BCS medium support this hypothesis: no peptides for YPO0995 could be detected under these growth conditions [Schrimpe-Rutledge AC and Adkins JN, unpublished data].

Like rhamnose and melibiose, several hypotheses have been advanced to explain why YP CO92 cannot metabolize glycerol. Analysis of the YP CO92 genome sequence revealed the presence of a 93 bp in-frame deletion in *glpD *that might account for this phenotype: *glpD *encodes aerobic glycerol 3-phosphate dehydrogenase, an enzyme that is essential for glycerol utilization [27], and this deletion likely disrupts protein function. Intriguingly, this same deletion appeared in every glycerol-negative strain in one culture collection whereas all glycerol-positive strains from the same collection contained an intact *glpD *[27]. There is a second annotated glycerol 3-phosphate dehydrogenase within the YP CO92 genome, *gpsA*, but the *gpsA *homolog in *E. coli *can only catalyze the transformation from dihydroxyacetone phosphate to glycerol 3-phosphate in an irreversible manner (Figure 3). A second defect occurs in the *glpFKX *operon and might contribute to the glycerol-negative phenotype as well: both the hypothetical protein *glpX *(YPO0089) and, more importantly, the glycerol kinase *glpK *(YPO0090) have also been disrupted by large deletions and are presumed to be pseudogenes. As with *glpD*, there is a second annotated glycerol kinase in the YP CO92 genome, YPO3312, but it is unknown whether YPO3312 can duplicate the function of YPO0090.

We saw slow growth for YP CO92 in both experimental measurements and model simulations in the absence of glycine and threonine, and the model predicts that this phenotype stems from insufficient supply of nitrogen. BCS medium does not contain an explicit source of nitrogen such as NH_4_Cl; therefore, YP CO92 most likely obtains elemental nitrogen through catabolism of one or more amino acids. Glycine, threonine, and serine can all interconvert. In turn, the breakdown of L-serine by L-serine dehydratase (YPO1771) or L-threonine by L-threonine dehydratase (YPO3896) leads to the direct formation of NH_3_. Simulations that exclude glycine and threonine from the *in silico *growth medium (serine is not a component of BCS medium) force nitrogen acquisition to occur through multi-step pathways that are less efficient, leading to slower growth. Providing supplemental nitrogen through sources such as NH_4_Cl, in contrast, leads to a faster *in silico *growth rate that varies with the uptake rate of the supplemental source.

It is well known that certain *Y. pestis *strains such as CO92 display methionine auxotrophy [25,26], and the model highlights the importance of the methionine salvage pathway to this phenotype. Several reactions in this pathway are not currently associated with any genes in YP CO92; however, if this pathway is absent, the byproduct S-methyl-5'-thioadenosine (MTA) would be generated during reactions that consume S-adenosyl methionine, but MTA itself would never be consumed or degraded in any reaction. Such a situation is identical to the formation of L-lactaldehyde during rhamnose utilization and results in the same thermodynamically impossible outcome. Furthermore, this pathway is fully annotated in other *Y. pestis *strains such as Pestoides F and Angola, suggesting that it is present in YP CO92 as well but that the associated genes remain to be identified. For these reasons, the model includes a set of non-gene-associated reactions that recycles MTA back into methionine via the methionine salvage pathway, and predicts that these reactions are essential in YP CO92.

## Conclusions

The analyses presented here concerning lysine, fatty acid, amino acid, rhamnose, melibiose, and glycerol metabolism help to illustrate how flux-balance analysis (FBA) of well-curated metabolic models can provide a context for interpreting experimental data. The crucial information provided by FBA is pathway utilization: whereas visual inspection of static pathway diagrams alone can indicate whether a certain pathway is present or absent in an organism, visual inspection cannot indicate whether a certain pathway carries flux and is therefore utilized. In contrast, FBA can provide this insight. For example, YP CO92 appears to have the ability to metabolize rhamnose because a complete set of genes and reactions connects imported rhamnose to central metabolism, but FBA analysis suggests that the absence of L-lactaldehyde degradation prevents this pathway from being utilized. Similarly, the gaps in lysine and fatty acid biosynthesis are readily apparent when genome annotation data is overlaid on static diagrams for the two pathways, but the model highlights the importance of these gaps - that they are essential reactions - since growth simulations cannot take place unless the gaps are filled. Looking forward, we anticipate that this reconstruction will serve as a platform with which to reconstruct the metabolism of other strains within the *Yersinia *genus and to integrate and interrogate -omics data sets generated for these organisms. Integrated models for other pathogens have already provided insight into important phenomena such as drug-target networks [34,35] and host-pathogen interactions [36]. Similar efforts for YP CO92 would further underscore the value that metabolic models bring to the interpretation of experimental data and likely generate additional important and testable hypotheses for this dangerous human pathogen.

## Methods

### Reconstruction approach

The YP CO92 model iPC815 was reconstructed via a four-step process [37,38]. The first step was to build a draft reconstruction. The complete list of metabolic genes in the YP CO92 genome [21] was assembled from the NCBI Reference Sequence database [39] downloaded on 1 May 2009 (RefSeq accession NC_003143). Next, we performed homology mapping between YP CO92 and *E. coli *K12 MG1655 and between YP CO92 and *Salmonella enterica *serovar Typhimurium LT2. The three bacteria are all closely-related enterobacteria and published reconstructions for both *E. coli *[8] and *S*. Typhimurium LT2 [9] already exist. Homologous metabolic genes in *E. coli *and *S*. Typhimurium were carried over to form the initial YP CO92 reconstruction. Homology was determined through one of the following: (1) a Smith-Waterman protein search [40] with a query of all curated metabolic genes from both models and a list of all predicted proteins in YP CO92 using SSearch [41]; (2) a DNA search for all curated metabolic ORFs in the *Y. pestis *CO92 genome using FASTA [41]; and (3) a translated protein search for all curated metabolic proteins in the YP CO92 genome by using tfasty [41]. A gene was considered shared if at least one of these three methods produced an alignment with a minimum of 75% sequence conservation over 75% of the query gene length. These cutoffs were chosen by comparing the published *E. coli *[8] and *S*. Typhimurium [9] metabolic reconstructions. With *E. coli *as the reference and *S*. Typhimurium as the query, these cutoffs led to approximately 75% overlap between the two models and a 2.5% false positive rate, defined here as the number of *E. coli *genes predicted to have homologs in *S*. Typhimurium but that did not carry over after manual curation.

With the final list of shared metabolic genes, we returned to the previously published metabolic reconstructions, this time focusing on the potential for shared reactions instead of shared genes. A reaction was considered shared if it had a complete gene-protein-reaction association (GPR) in the YP CO92 model. A complete GPR is one in which all genes required to catalyze a particular reaction are present. For example, a GPR in the *E. coli *or *S*. Typhimurium models catalyzed by gene 1 AND gene 2 was ported to the YP CO92 model only if YP CO92 was deemed to have homologs for both gene 1 and gene 2. If neither or only one of the two genes was present, the reaction was not considered shared and was not ported.

Reactions known to be spontaneous in both the *E. coli *and *Salmonella *models were also added to the YP CO92 model, completing the draft YP CO92 metabolic reconstruction. Reactions without gene-protein associations ("orphan" reactions) were not ported.

The second step was model refinement. We searched approximately 2700 publications in PubMed using the keywords "Yersinia" and "metab*" to collect experimental data for as many genes in the draft reconstruction as possible. Our search focused on the YP CO92 strain in particular, but we also noted any relevant data from any species within the *Yersinia *genus. We used the PATRIC [13], KEGG [10], and other databases in our search as well. Genes, proteins, and reactions in the draft reconstruction were updated according to the experimental data uncovered during these searches. All reactions were then mass- and charge-balanced.

The third step was to convert the refined reconstruction into a mathematical format suitable for computation, the stoichiometric matrix (S-matrix), and to develop explicit mathematical equations for the biomass objective functions (BOF). The model contains two BOFs that simulate biomass formation at 25-28°C and 37°C. Both are derived from similar BOFs constructed for the published *Y. pestis *91001 metabolic model [7] and modified to incorporate additional lipid composition and lipopolysaccharide (LPS) data as follows. First, the model contains explicit biosynthesis pathways for the 14:0 (including 3'-OH-14:0), 16:1, 16:0, and 18:1 fatty acid chain lengths since these four dominate the fatty acid composition in *Y. pestis *at 27-28°C [15,42]. We could not find fatty acid composition data for *Y. pestis *at 37°C, but we assume that this distribution also holds at 37°C based on data from *Y. enterocolitica *at 37°C [18]. Accordingly, we subdivided cardiolipin (clpn), phosphatidylethanolamine (pe), and phosphatidylglycerol (pg) such that each of the three contains all four acyl groups in both BOFs. Second, the LPS differs between the two temperatures: in *Y. pestis *KIM218, another biovar Orientalis strain, galactose is present as one of the monomer units in the core oligosaccharide at 25°C but not 37°C [17]. Moreover, the core oligosaccharide at both 25°C and 37°C contains one 3-deoxy-D-manno-oct-2-ulosonic acid (Kdo) and one D-glycero-D-talo-oct-2-ulosonic acid (Ko) rather than two Kdo residues, which is the more common structure in Gram-negative bacteria [16,17]. The composition of the acyl chains attached to the core oligosaccharide also varies with temperature [17]. *Y. pestis *CO92 LPS does not contain O-antigen at either temperature [43].

The fourth step was model evaluation and debugging. Critical gaps preventing *in silico *production of biomass precursor metabolites were filled by identifying and adding to the model enzymes with the potential to catalyze the missing reaction(s). These candidates were gleaned from primary literature, genome annotation, and database sources such as KEGG and PATRIC. A similar pathway analysis procedure was used to ensure that the model reproduced known YP CO92 amino acid auxotrophies [26].

The model (S-matrix) was implemented in Matlab and flux balance analysis (FBA) performed using the COBRA Toolbox and glpk solver [44]. The S-matrix is an *m *by *n *matrix containing *m *metabolites and *n *reactions. The relationship between reactions and the concentration time derivatives is:

$$\frac{dx}{dt}=S\cdot v$$

where *x *and *v *denote the vector of metabolite concentrations and reaction fluxes, respectively. Steady-state FBA is performed by solving the following linear optimization problem:

$$\begin{array}{c} \text{maximize(}c^{\text{T}}\cdot v\text{)}\\ \text{subject}\;S\cdot v=0\;\text{and}\;\text{lb}<v<\text{ub} \end{array}$$

The reaction fluxes are constrained by thermodynamic and reaction kinetics and lie between lower (lb) and upper (ub) bounds. The optimization vector (**c**) is a zero-vector except for one element that has a value of one and corresponds to the reaction to be optimized, which in the model is one of the two BOFs. Model constraints during simulations were set by either experimental data collected as part of this study (Additional File 1) or, if certain uptake rates or other experimental data were not available, by setting the lower bound to the initial concentration of that particular compound in the experimental BCS growth medium [24]. A minimal medium consisting of a carbon, nitrogen, phosphorus and sulfur source plus unconstrained amounts of Ca^2+^, Cl^-^, Fe^2+^, H_2_O, H^+^, K^+^, Mg^2+^, Mn^2+^, O_2_, PO_4_^3-^, SO_4_^2-^, pantothenate, and thiamin was used during simulations to investigate the ability of different sources of the four elements to support *in silico *growth (Additional File 1). These chemicals are present in BCS medium [24]. To examine gene essentiality, genes were removed from the model and the resulting flux through the BOF assessed. Zero flux through the BOF was taken to be an indicator of an *in silico *essential gene.

The reconstruction has been made available in sbml format as Additional File 3.

### Cultivation

*Y. pestis *CO92 was grown in a chemically defined BCS medium [24] in which neutral pH (7.2) was maintained by the addition of 50 mM of morpholinopropanesulfonic acid (MOPS) as described previously [45]. The medium contained 4 mM CaCl_2 _and one of the carbon sources at a concentration of 0.2%. Bacterial cultures were grown at 26°C in Erlenmeyer flasks aerated at 200 rpm. The first culture was grown during daytime, and then overnight culture was initiated at an optical density at 600 nm (OD_600_) of 0.1. Both the first and second cultures contained 0.2% potassium gluconate as a carbon source [45], and the overnight culture typically reached an OD_600 _of ~3.0. The third culture was obtained from bacteria grown overnight, washed twice with 33 mM potassium phosphate buffer pH 7.0, and inoculated into fresh media containing one of the eleven tested carbon sources to an initial OD_600 _of 0.05. Aliquots from cultures were taken at 0, 2, 4, 6, 8 and 10 hours time points to measure OD_600 _and to prepare samples for metabolite identification and uptake/secretion measurements. These samples were obtained by centrifugation of 1.5 ml of bacterial culture at 10,000 rpm for 2 minutes at 4°C followed by filtration of the supernatants through a 0.2 μm low binding polyethersulfone membrane filter (Corning Life Sciences, Lowell, MA). Samples were then immediately frozen and stored at -80°C.

### ^1^H NMR Spectroscopy and Metabolite Quantification

540 μL of spent media extracts were added to 60 μL of 5 mM 2,2-dimethyl-2-silapentane-5-sulfonate (DSS) in 99.9% deuterium oxide (D_2_O) in 5-mm NMR tubes. DSS is used as an internal standard and to provide a ^1^H chemical shift reference at δ 0.00 ppm. ^1^H NMR spectra were acquired on a Varian INOVA-600 MHz NMR spectrometer (Varian Inc., Palo Alto, CA) at 298 K, using a triple resonance 5-mm HCN salt-tolerant cold probe. A one-dimensional NOE pulse sequence adapted from the two-dimensional Varian tnnoesy was used. For each sample, 96 transients were collected into 64 K data points using a spectral width of 7225.4 Hz with a relaxation delay of 1.0 s, an acquisition time of 4.00 s, and a mixing time of 100 ms. Spectra were processed using Chenomx 6.1. A 0.5-Hz line-broadening function was applied to all spectra prior to Fourier Transformation (FT) and baseline correction. The profiler module of Chenomx was used to identify and quantify metabolites.

## Authors' contributions

PC, JAL, and DRH constructed the model. SC performed the experimental work on YP CO92. KA performed NMR measurements. KA, PC, SC, VM, CA and JNA analyzed the data. CA, JNA and BOP coordinated the project. All authors contributed to writing and approve the manuscript.

## Supplementary Material

Additional file 1 **Model details**. A. Table of contents. B. Gene list. C. Metabolites. D. Reactions and their associated genes (if any). E. Biomass objective functions. F. Comparison between iPC815 and the YP 91001 model of Navid, et al. G. Growth simulations on different carbon sources. H. Growth simulations on different nitrogen sources. I. Growth simulations on different phosphorus sources. J. Growth simulations on different sulfur sources. K. Individual components in BCS growth medium. L. Substrate uptake and secretion rates used to constrain model simulations. Click here for file

Additional file 2 Gene essentiality. A. List of genes predicted to be essential in YP CO92. Homologous genes in *E. coli *and *Salmonella *Typhimurium LT2 that were predicted to be essential by the *E. coli *iAF1260 model [8] and the *Salmonella *Typhimurium LT2 consensus model [9] have been listed as well. B. List of genes predicted to be essential in *E. coli *only (from [8]). C. List of genes predicted to be essential in *S*. Typhimurium only [9]. Click here for file

Additional File 3 **The iPC815 model in SBML format**. Click here for file
